# Engineering C1q single-chain globular head variants for enhanced IgM binding

**DOI:** 10.1186/s12915-026-02570-2

**Published:** 2026-03-12

**Authors:** Maria Magdalena John, Anton Barthel, Vanessa Hawlin, Gordana Wozniak-Knopp, Renate Kunert

**Affiliations:** 1 Institute of Animal Cell Technology and Systems Biology, Department of Biotechnology and Food Science, BOKU University, Vienna, Austria; 2 Institute of Molecular Biotechnology, Department of Biotechnology and Food Science, BOKU University, Vienna, Austria

**Keywords:** C1q variants, IgM, C1q, Protein interaction, Protein engineering, Yeast surface display

## Abstract

**Background:**

The initiation of the classical complement pathway begins with the binding of the globular head of complement component 1q (C1q) to antigen-bound immunoglobulin M (IgM). To investigate the binding mechanism and sites of C1q, a single-chain protein mimetic of the globular head of C1q and variants thereof were designed.

**Results:**

Two approaches were used to generate single-chain globular head C1q variants containing single point mutations potentially altering IgM/C1q binding. The rational protein engineering approach aimed to increase surface charge, considering the negatively charged IgM Cµ3 region and positively charged C1q globular heads. Further, a library of 646 variants with single point mutations in the C1q B-chain loops was designed and expressed using yeast surface display. Three rounds of panning in IgM-coated plates yielded twenty-eight sequenced yeast colonies. The His-tagged wild type variant and six of nine selected variants were stably expressed in Chinese hamster ovary cells and purified using immobilized-metal affinity chromatography. All variants were tested for IgM interaction in competition with serum-derived C1q and in a complement activation assay to evaluate the C1q competition potential of the single-chain globular head proteins.

**Conclusions:**

Expression levels differed among the globular head C1q variants, and SDS-PAGE analysis revealed variations in migration mobility, suggesting conformational differences. Four variants showed enhanced IgM binding compared to the wild type variant indicated by improved C1q displacement in the competitive interaction assay. These results were further supported by an advanced complement activation assay, where these variants significantly inhibited complement activation. These findings underpin the critical role of specific amino acids for IgM/C1q interaction and highlight the potential of engineered C1q as a potent inhibitor or activator of the classical complement cascade.

## Background

The complement system is a crucial component of the innate and the adaptive immune response, playing a significant role in the defence against pathogens [1]. The classical pathway of complement activation is predominantly triggered by immunoglobulins M and G (IgM and IgG) upon binding to their specific antigens [2]. The first component of this pathway, C1q, recognizes various structures, including the constant regions of immunoglobulins, thereby initiating the complement cascade [3].

The structural activation model suggests that IgM, upon antigen binding, undergoes significant conformational changes. Specifically, the flexible Cµ2 region transitions from a planar and highly mobile conformation to a bent and rather fixed structure [4, 5]. These structural changes enable the exposure of a previously cryptic motif within the Cµ3 domain, the predicted binding site for the globular heads of C1q [6]. Once the multimeric C1 complex binds to the immunoglobulin, it triggers a proteolytic cascade, resulting in a cytolytic effect which eliminates the foreign invader [7].

In our previous study [8], we investigated the interactions between various recombinant IgMs and C1q derived from human serum (sC1q), or recombinantly expressed single-chain variants of the trimeric globular region of C1q, based on the concept of Moreau et al. [9].

In the present study, newly defined globular head C1q (ghC1q) variants with single point mutations in the B-region of the globular head of C1q were developed using two different approaches: rational protein engineering and random mutagenesis, followed by yeast surface display selection and screening.

In surface display techniques, the isolation of binding proteins from libraries is typically achieved using screening tools based on the principle that each mutant protein is linked to its genetic code. This allows for the identification of proteins that bind to the target. In yeast surface display, the protein of interest is most commonly covalently fused to a cell wall protein and displayed on the cell surface [10].

In contrast, rational (knowledge-based) protein engineering aims to exploit information on protein structure to predict its function or binding properties and is often complemented by computational models and simulations. This approach aids hit identification from a theoretically smaller repertoire and therefore reduces the required library size [11].

Finally, the identified ghC1q variants were expressed in Chinese hamster ovary (CHO) cell cultures, purified, and analysed for their binding behaviour to hexameric IgM in a competitive analytical approach. The aim of this investigation is to identify relevant and beneficial mutations that enhance IgM/C1q binding and to gain a deeper understanding of the structural and functional dynamics of specific residues on the globular head of C1q. This research could potentially pave the way for the development of improved recombinant C1q.

## Results

### ghC1q variants designed by rational protein engineering

The globular head C1q single-chain mimetic was used as a starting material for engineering of C1q [8, 9]. Two approaches were applied to design variants of the ghC1q with only a single point mutation in the B-chain protein sequence. Two variants were defined by rational protein engineering. As C1q is reported to be conserved across different species, the D110N variant was selected based on a multiple sequence alignment of the C1q B-chain of human (UniProtKB P02746 [12]) with mouse (UniProtKB P14106 [13]), rat (UniProtKB P31721 [14]), and bovine (UniProtKB Q2KIV9 [15]). Since the positively charged arginine residues R108 and R109 have been consistently reported to play a critical role in the IgM/C1q interaction, the adjacent negatively charged aspartic acid D110 was changed to uncharged asparagine, which is found in mouse and rat [16–18]. The F178R variant is based on the publication by Sharp et al. [4]. They identified F178 on the B-chain as a potential key residue for IgM/C1q interaction by modelling approaches. Because of the reported electrostatic forces-guided interactions between the residues of IgM and C1q, the positively charged arginine was introduced to increase the surface charge.

### ghC1q variants as a result of yeast surface display

To obtain ghC1q variants with improved binding properties to IgM, a yeast surface display approach was applied. Instead of using a fully random library, the library design focused on the mutagenesis in C1q B-chain, known to form the preferred IgM binding region [16–18]. To preserve the structure of the head, only the variable regions, known as loops, were mutated. For better comparison, each variant contained only a single point mutation, allowing us to trace back the specific effect of each amino acid substitution. Therefore, the mutations were placed in the four flexible loops of the C1q B-chain. Each of the 34 selected amino acids was substituted with every individual amino acid of the remaining 19 amino acids, resulting in a library of 646 variants, each containing a single point mutation compared to the wild type (WT).

**Fig. 1:**
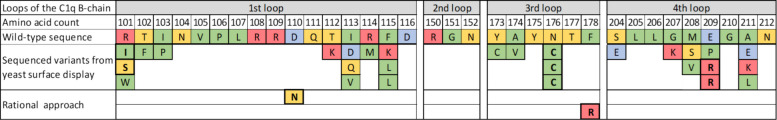
Sequencing results of the yeast library output after IgM panning and amino acid exchanges designed by rational engineering approach. Amino acid abbreviations in one-letter code. Amino acid colour code: blue = negatively charged, red = positively charged, yellow = uncharged polar, green = nonpolar

After transformation of the yeast cells, cultivation and induction, the yeast culture was stained and analysed by flow cytometry. Approximately 60% of the cells showed a positive signal for the C-terminal c-Myc fusion tag, indicating that the ghC1q variant was expressed on the yeast surface. The yeast culture was enriched for better IgM binders by panning on IgM-coated 96-well plates. After three rounds of selection, the yeast cells were plated, and 28 colonies were sequenced. The results are shown in Fig. 1.

Interestingly, one region in the first loop, and the entire second loop were found without mutations, suggesting that these regions are best kept conserved to support IgM/C1q interaction.

The positively charged residues R101 and R114 were changed to uncharged polar and nonpolar amino acids, thereby shifting the surface charge of this region to less positive. To cover both exchanges and to allow a direct comparison of the R101 mutations, the R101I and R101S variants were selected for further investigation. Isoleucine was selected over tryptophan to circumvent the critical sterically driven change by the introduction of a bulky aromatic amino acid.

In the case of T112, F115, and G207, an uncharged amino acid was changed to a neutral or positively charged amino acid. To gain more information about the influence of electrical charge, the variants T112K and F115K were selected for further investigation. Conversely, the amino acids I113 and S204 were changed from uncharged nonpolar or polar to negatively charged or neutral amino acids. While the side chain of I113 is nearly completely buried, S204 is solvent-exposed, which is why the variant S204E was chosen for further characterization.

For the amino acid E209, a negatively charged amino acid was exchanged for a neutral or positively charged one, resulting in an overall more positively charged surface region. Here, the variant E209R featuring charge reversion was selected for further investigation. For the amino acid A211, the uncharged amino acid was exchanged for neutral, positively, and negatively charged amino acid, showing no clear trend. Therefore, it was not considered for further studies.

No change of charge was observed for the amino acids T102 and I103 in the first loop, any of the amino acid changes in the third loop, or the amino acid M208 in the fourth loop and therefore they were not considered for the expression in soluble form. The exception was N176C, which was isolated three times from the 28 yeast output colonies and was deemed worthy of further investigation.

In total, seven ghC1q variants were selected for expression in CHO cells, and the respective plasmids were created via site-directed mutagenesis, namely R101I, R101S, T112K, F115K, N176C, S204E and E209R.

### Production and purification of the ghC1q variants

The wild type and its variants were cloned into their respective vectors, and stable CHO-K1 cell lines were created. Culture supernatant was collected daily from a semi-perfusion process that was conducted over a period of twelve days.

The cultures of WT and D110N, as well as F178R, generated by rational mutations, reached cell densities of 10–30 × 10^6^ cells/ml with titers of 100 µg/ml for WT (180 ml), 78 µg/ml for D110N (180 ml) and 125 µg/ml for F178R (160 ml). The production rate of the WT was the highest, with an average of 8.9 pg/cell/d, while the production rate for D110N and F178R was lower, with 2.1 pg/cell/d and 2.5 pg/cell/d, respectively.

The variants extracted from the yeast library approach reached cell densities between 30 and 60 × 10^6^ cells/ml. The titers of the culture supernatants for the variants R101I, T112K, F115K and S204E were below the detection limit in quantitative biolayer interferometry measurements using HIS1K sensors. The titers of the measurable supernatants were 25.8 µg/ml (310 ml) for R101S, 17.0 µg/ml (200 ml) for N176C and 40.0 µg/ml (315 ml) for E209R. The average production rate was 1.0 pg/cell/d, 0.4 pg/cell/d and 1.3 pg/cell/d, respectively. In the Western blot a positive signal for R101I was detected, and consequently the variant was also purified from 520 ml culture supernatant.

The purification process was optimized for the WT variant by adjusting the imidazole and salt concentrations in the equilibration and elution buffers to minimize unspecific binding to the column [19]. The primary objective was to achieve a high level of purity with a one-step protocol, eliminating the need for an additional chromatography step, such as size exclusion chromatography. Depending on the titer and cultivation volume, different amounts of each variant could be recovered from the supernatants. The final amounts ranged between 260 µg and 1.5 mg, with concentrations between 40 µg/ml and 937 µg/ml determined with quantitative biolayer interferometry. Despite the ActiPro medium was beneficial for the growth of the cultures, the titer and productivity of the variants cloned in the pcDNA3.1 vector were lower compared to the variants cloned in the pL vector.

**Fig. 2:**
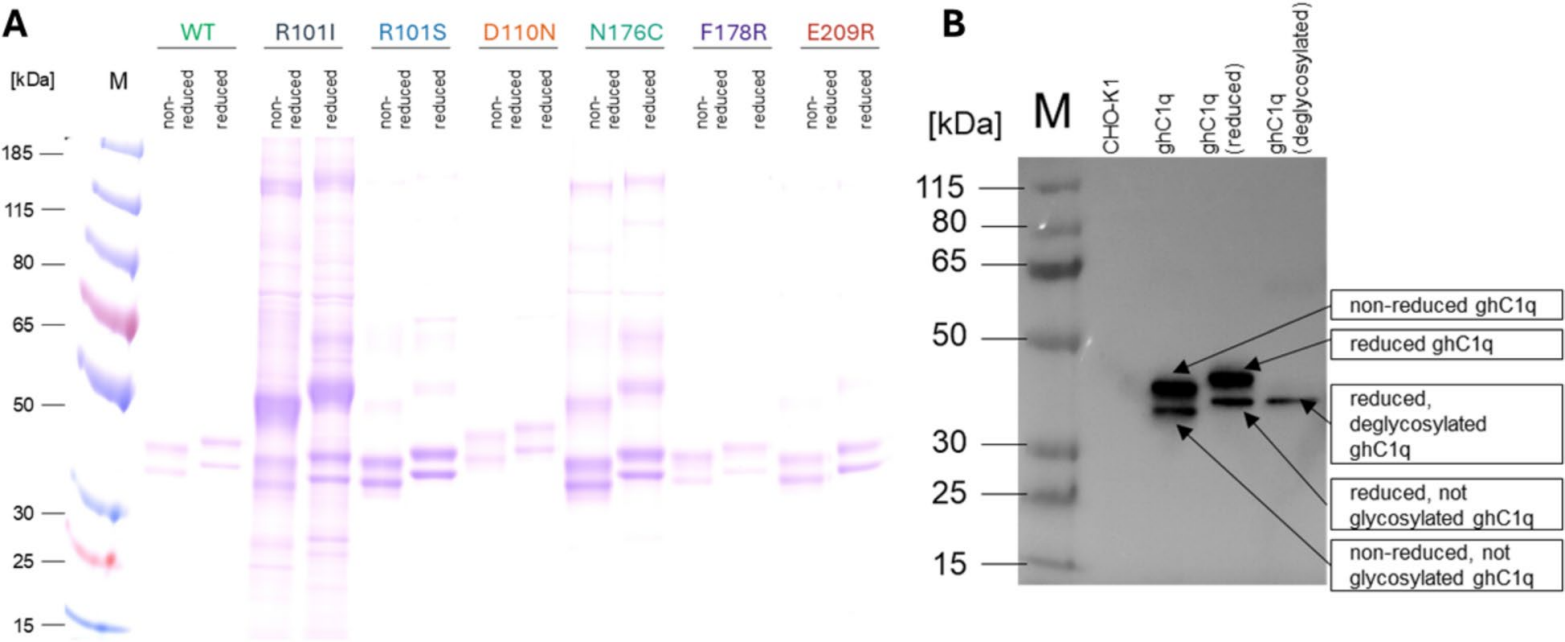
Analytical results of the purified ghC1q variants. **A** SDS-PAGE of all ghC1q variants under non-reducing and reducing conditions. **B** Western blot of the ghC1q WT variant stained with 6xHis-tag antibody. CHO-K1 host cell line supernatant was used as negative control. WT sample is analysed under non-reducing and reducing conditions and following PNGase F-mediated deglycosylation in reducing conditions

The purified ghC1q variants (WT, R101I, R101S, D110N, N176C, F178R, E209R) were analysed by SDS-PAGE and Western blot as shown in Fig. 2. These methods confirmed that the ghC1q variants ranged from 30 to 50 kDa, consistently with the theoretical molecular weight of 47 kDa. Each variant exists as a glycosylated and a non-glycosylated form with N-linked glycosylation on the C1q A-chain [20]. The higher band seen in the non-reduced and reduced lanes corresponds to the glycoprotein, whereas the lower band represents the non-glycosylated form. These findings from the SDS-PAGE were confirmed in the Western blot (Fig. 2B), where the WT variant was deglycosylated using PNGase F and the treatment resulted in a single band of the same height as the lower band of the reduced ghC1q sample.

A comparison of the different variants in the SDS-PAGE (Fig. 2A) shows that, with the exception of the D110N variant, all other variants show wild type like mobility in the gel. However, both the glycosylated and non-glycosylated form of the D110N variant show reduced mobility compared to the WT.

In evaluating the purity of the variants, it is notable that the WT, D110N, F178R and E209R variants exhibit no discernible impurities. However, the R101S variant displays trace impurities, particularly at approximately 50 kDa. In contrast, the N176C, and most prominently, the R101I variant, show a substantial presence of impurities. The degree of impurities is inversely correlated with the overall titer of the variant in the supernatant and its production rate. Despite optimization of the purification process, the single step purification protocol demonstrates limitations when applied to variants with low production titers, leading to elevated impurity levels.

### Modelling and prediction of ghC1q variants

All ghC1q variants selected from the protein engineering and yeast display approaches and expressed in CHO cells were modelled with PyMOL and the electrostatic calculations were performed with the Adaptive Poisson-Boltzmann Solver (APBS). With the substitution of one amino acid of the wild type, PyMOL suggests different numbers of rotamers with percentages for each rotamer. The rotamers differ in the angle of the newly introduced residue and the percentage represents the probability of the rotamer. The number of rotamers and therefore the percentual distribution depends on the position, size and space of the exchanged amino acid. The lower the number of rotamers, the higher the percentage for each rotamer, indicating a limited space for the new residue to “settle” in the protein configuration. The R101I, R101S and N176C variants each had three rotamers, with the highest percentages of one rotamer ranging from 71.7% to 79.8%. The D110N variant had nine rotamers with the highest percentage of 46.8%. In contrast, the F178R and E209R variants showed 22 and 24 rotamers with the highest percentage of 17.1% and 17.3%, respectively, suggesting several conformational possibilities for the exchanged residue to fit in the protein structure. For the APBS analysis, the rotamer with the highest percentage was used.

**Fig. 3:**
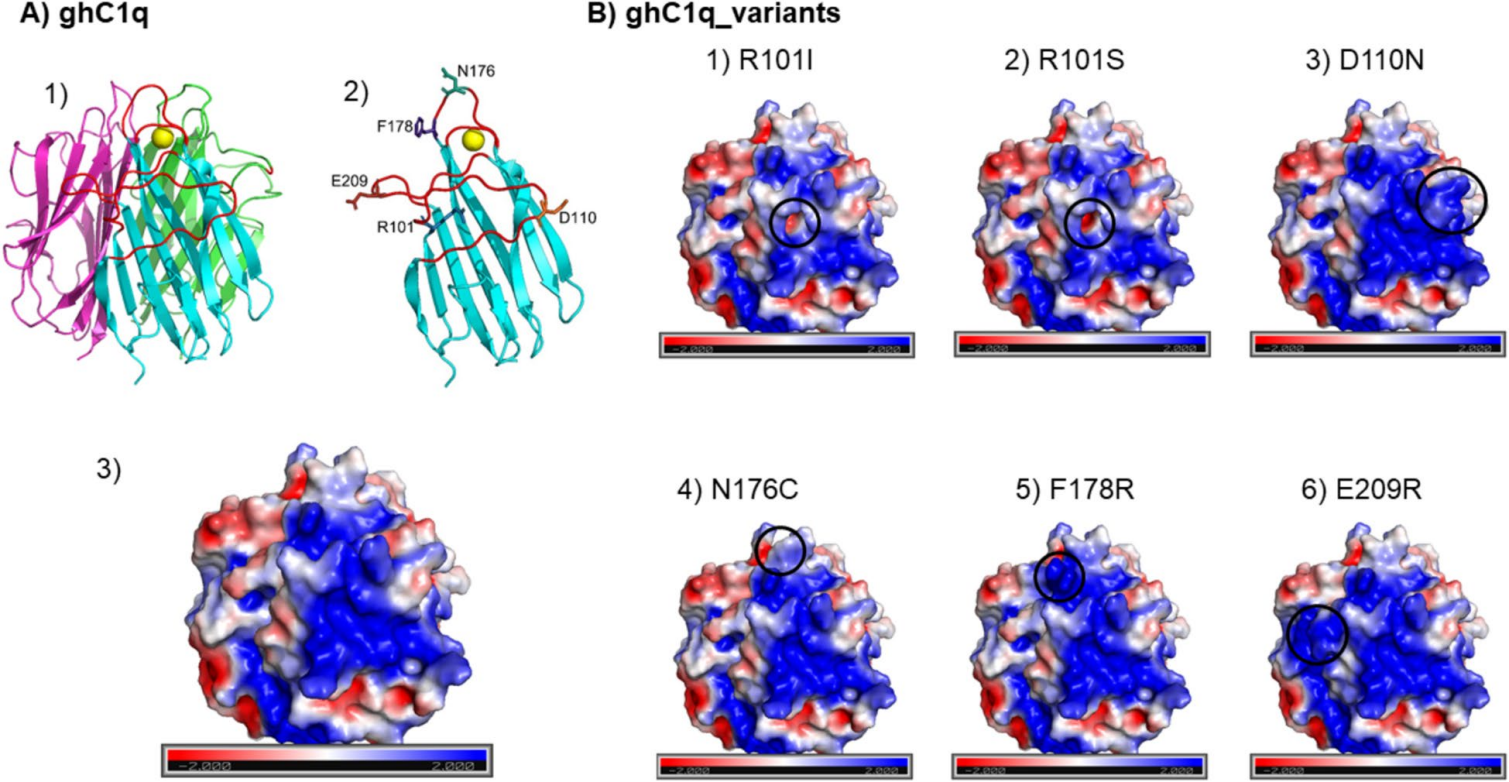
ghC1q protein structures. A1) ghC1q as cartoon structure. A-chain is pictured in green, B-chain is pictured in cyan with the mutated loops in red and C-chain is pictured in pink. The calcium ion is pictured in yellow. A2) globular part of the B-chain with the mutated loops in red and the exchanged amino acids indicated with residue. A3) ghC1q WT modelled with APBS solver analysis in PyMOL. Negatively charged sites indicated by red colour, positively charged sites indicated by blue colour. B) ghC1q variants modelled with APBS analysis in PyMOL. The exchanged amino acid and the resulting change of charge is marked by a black circle

Figure 3 illustrates the models created with PyMOL of the different variants. The models demonstrate the proposed distribution of charge on the protein surface. The change in charge in the R101I and the R101S variants is from positive to neutral, causing a net negative charge in this region. The WT is colored blue at this site, indicating a positive charge, while the R101I variant is lighter red at this site, indicating a slightly negative charge. The R101S variant exhibits an even more negative charge compared to the R101I variant, as illustrated by the dark red coloration in Fig. 3B. The D110N mutation results in a shift of the local charge from negative or neutral to positive, rendering the entire side of the B-chain uniformly positively charged site. The mutated amino acid of the N176C variant is located on the apical side of the protein, and while the electrical charge remains rather unchanged, the smaller van der Waals radius of cysteine compared to the asparagine leads to a narrower tip of the B-chain. In comparison, the mutation F178R adds a new positively charged bulge close to the apex. The mutated amino acid of the E209R variant is located on the side of the B-chain, in proximity to the C-chain. Exchanging the negatively charged amino acid for a positively charged one shifts the entire amino acid environment from slightly negative and neutral to completely positive.

### Competitive interaction assay

The ability of ghC1q variants to bind to hexameric IgM was measured indirectly using a competitive IgM/sC1q interaction assay. This assay format was chosen due to the fast on- and off-rate of the wild type ghC1q molecule, which precludes direct kinetic measurement of binding to IgM using techniques such as surface plasmon resonance or biolayer interferometry [8]. Instead, the displacement of sC1q from IgM by the co-incubation of individual ghC1q variants and C1q derived from human serum indicates the binding capacity of the ghC1q variants to IgM.

**Fig. 4:**
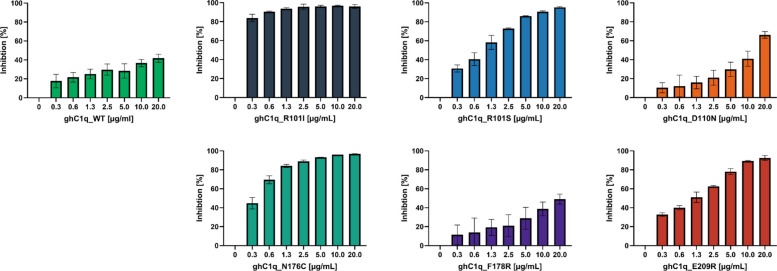
Competitive interaction assay of sC1q in presence of the ghC1q variants determined in 96-well plates coated with hexameric IgM. Standard deviations were determined from two parallel assays

In the assay, a 96-well plate was coated with hexameric IgM, and the ghC1q variants were serially diluted. A constant amount of sC1q was added to each well, and the resulting mixture was used as the sample. IgM/sC1q binding was detected using a labelled polyclonal anti-C1q antibody (Fig. 4). The x-axis of the graphs represents increasing concentrations of the respective ghC1q variant. The measured optical density values were normalized to the no inhibition control (sC1q only), resulting in inhibition percentages and displayed as bars. In the absence of ghC1q variants, no inhibition is observed, as only sC1q is present in the sample. In contrast, full inhibition is achieved at the highest concentration of the ghC1q variant, when the same amount of sC1q is present in the sample.

The WT variant exhibited the lowest maximum inhibition, which was below 50% even at the highest concentration of 20 µg/ml used here, whereas, the R101I variant demonstrated the strongest inhibition, with 84% inhibition at the lowest concentration of 0.3 µg/ml ghC1q. The remaining variants exhibited inhibition potential that ranged between the WT reference and the highest inhibition observed with R101I. It is evident that all variants inhibit sC1q and show a concentration-dependent response, with higher concentrations of ghC1q resulting in higher inhibition.

The WT variant exhibited inhibition ranging from 18 to 42% across the tested concentrations of 0.3–20 µg/ml. The D110N and F178R variants showed slightly higher inhibition compared to the WT variant. Specifically, the D110N variant achieved 66% inhibition at 20 µg/ml ghC1q, and a minimum inhibition of 10% at 0.3 µg/ml ghC1q. The F178R variant reached 49% at 20 µg/ml ghC1q, decreasing to 11% at 0.3 µg/ml.

In contrast, the R101I variant achieved 97% inhibition with 10 µg/ml ghC1q, which only slightly decreased to 84% inhibition with 0.3 µg/ml ghC1q. The N176C variant exhibited 97% inhibition at 20 µg/ml ghC1q, gradually decreasing to 45% at 0.3 µg/ml. The R101S and E209R variants exhibited the highest levels of inhibition at a concentration of 20 µg/ml, with values of 95% and 93%, respectively. However, when these variants were diluted, the inhibition dropped to 31% and 33% at a concentration of 0.3 µg/ml ghC1q.

In conclusion, the D110N and F178R variants showed no higher inhibition compared to the WT reference, while the R101I, R101S, N176C and the E209R variants demonstrated substantial competition of the IgM/sC1q interaction.

### Competitive complement activation assay

The inhibitory effect of ghC1q variants on complement activation was determined by a competitive assay. To achieve a detectable effect of the added ghC1q variants, the assay protocol was carefully adapted from a published complement activation assay [21, 22]. Adjustments included balancing the background signal from C1q-depleted normal human serum by reducing its concentration, optimizing the sC1q concentration to generate a measurable signal, adjusting the salt concentration to ensure signal stability, and reducing the incubation time to prevent the enzymatic cleavage reaction of the C4 complex reaching the plateau phase.

**Fig. 5:**
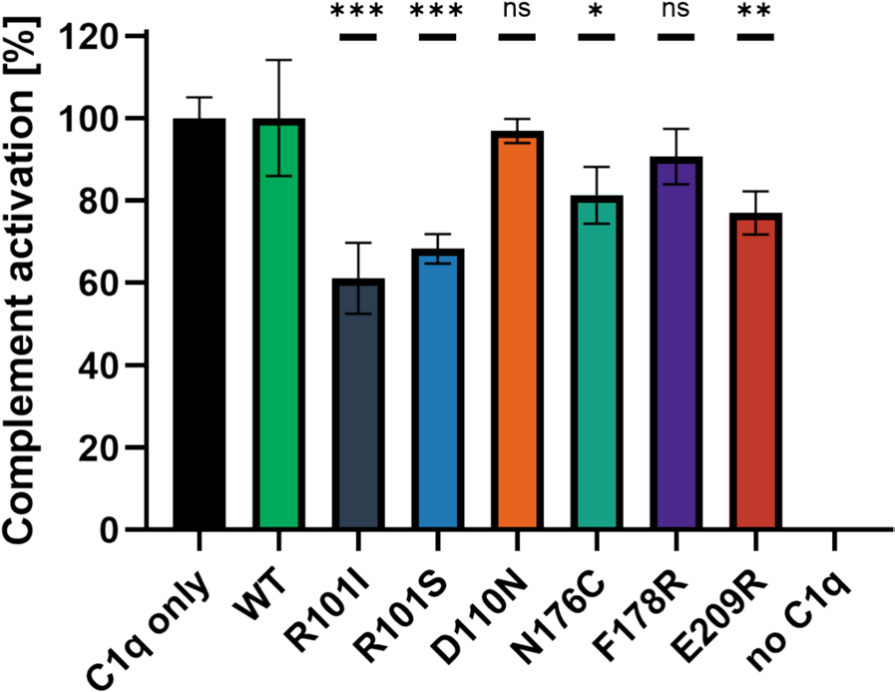
Competitive complement activation of sC1q was determined in the presence of the ghC1q variants in 96-well plates coated with hexameric IgM. Standard deviations were calculated based on eight measurements (four individual assays, each comprising two technical replicates). Statistical significance was assessed using the Mann–Whitney U test to compare the WT variant with the respective mutant ghC1q variant. P values: * *p* < 0.05, ** *p* < 0.01, *** *p* < 0.001, ns = not significant

Hexameric IgM-coated plates activated the complement pathway using C1q-depleted normal human serum in combination with sC1q. C4b levels were measured as readout to determine IgM/sC1q related complement activation, while the ghC1q variants served as competitors for sC1q. A negative control was established by omitting sC1q, the positive control was prepared with sC1q only, excluding any ghC1q variant. All the ghC1q variant samples were normalized to the controls. The results are shown in Fig. 5.

The WT, D110N and F178R variants did not significantly reduce the C4b-releasing potential of sC1q. In contrast, the R101 variants demonstrated substantial inhibition of complement activation and the N176C and E209R variants exhibited weak but statistically significant inhibition in the evaluation of eight replicates. The concentrations of sC1q and ghC1q variants were set at 0.25 µg/ml and 10 µg/ml, respectively, corresponding to a 40-fold (*w/w*) excess of the ghC1q variant. This relatively high concentration of ghC1q variant was necessary due to the fast on- and off-rate in the binding kinetic of the ghC1q mimetics [8].

## Discussion

In this project we have explored the interaction between hexameric IgM and single-chain globular head C1q variants. The ghC1q variants were created through two approaches: rational protein engineering, and panning of a yeast display library, where each variant differed from the WT ghC1q in a single point mutation. The variants were expressed in CHO cells, purified and tested in competitive interaction and complement activation assays. The results of these experiments, as well as the impact of the residue substitution, are discussed here.

### Functional and structural impact of single point mutations in ghC1q variants

The capacity of the globular head C1q variants to interact with IgM was evaluated using competitive interaction and activation assays (Figs. 4, and 5). Thereby ghC1q variants prevent the binding of C1q derived from human serum to IgM by occupying its binding sites. Table 1 summarizes the ghC1q variants according to their key properties.

**Table 1:** Summary of the expression, purification, and activity properties of ghC1q variants

Variants	Engineering approach	Titer [µg/ml]	Production rate [pg/cell/d]	Purity in SDS-PAGE	Competitive interaction assay	Competitive activation assay
					Interpolated IC_50_ [µg/ml]	Complement activation [%]
WT		100	8.9	High	> 20	100
R101I	Yeast display	< 13	< 0.3	Very low	< 0.3	61
R101S	Yeast display	26	1.0	Moderate	0.85	68
D110N	Sequence alignment	78	2.1	High	12.6	97
N176C	Yeast display	17	0.4	Low	0.34	81
F178R	Model-based	125	2.5	High	21.1	91
E209R	Yeast display	40	1.3	High	1.08	77

Based on literature and interaction models, charged residues were predominantly selected as the IgM/C1q interaction is described as primarily ionic in nature [4, 16, 17, 23]. For the R101I, R101S, D110N, F178R and E209R variants, the overall surface charge of the ghC1q variants was changed compared to the WT. In line with the initial hypothesis that increasing the surface charge would enhance IgM/C1q interaction, the D110N and F178R variants from the rational approach, and the E209R variant from the yeast library screening, were tested.

The D110N variant was designed based on the multispecies C1q sequence alignment, and it was exchanged as it is found in mouse and rat [13, 14]. The amino acid exchange increases the positively charged area near the conserved residues R108 and R109 [16–18] with the intention to enhance electrical attraction to IgM. It is important to note that this mutation has been observed to impact the mobility of the protein in SDS-PAGE (Fig. 2A). This finding suggests that the protein may have adopted an alternative conformation, and the negative charge of the D110 residue is essential for maintaining the molecular configuration of the ghC1q. It is also noteworthy that no yeast clones with a mutation in the region of the D110 residue were identified (Fig. 1), thereby supporting the conclusion that modifying this region will not yield superior IgM binders.

The F178R variant is based on published data [4], which claims that the interaction of F178 with the DLPSP loop of IgM is essential for the IgM/C1q binding. The decision to substitute this phenylalanine with arginine was based on the observation that other species-conserved residues in ghC1q that interact with IgM are arginine (R108, R109 [16–18] and R150 [4]). Consequently, it was deduced that arginine appears to be advantageous and fundamental in this interaction, thus prompting the implementation of the mutation.

However, the competitive interaction assay indicated for the D110N and F178R variants a half maximal inhibitory concentration (IC_50_) substantially higher than that of the other mutated ghC1q variants. In addition, the competitive activation assay yielded no significant results in comparison to the WT variant (Table 1). These results suggest that the residues D110 and F178 are not involved in the IgM/C1q interaction.

For the E209R variant, derived from unbiased yeast display, the amino acid exchange removes a negative surface charge, thus rendering a positively charged area on the protein surface. The variant binds IgM and causes sC1q displacement, with inhibition of IgM/sC1q binding (IC_50_ of 1.08 µg/ml, Table 1) and significant prevention of complement activation. The E209 residue is not part of the generally accepted residues assumed to be involved in IgM/C1q interaction [4, 16–18].

The change from a positively charged amino acid to a nonpolar or uncharged polar one, as observed in the R101 variants, results in a negative surface charge. This contradicts the initial hypothesis that a positively charged surface would be beneficial for IgM/C1q interaction. However, the present study has demonstrated that both amino acid exchanges of R101 contribute positively to the inhibitory capacity of the ghC1q variant, despite the findings of other studies that arginine plays a crucial role in the IgM/C1q binding process [16, 18].

The R101I variant demonstrated the most potent inhibition of sC1q, with a calculated IC_50_ of < 0.3 µg/ml (Table 1) in the competitive interaction assay. Furthermore, the variant demonstrated a significant capacity to inhibit complement activation. It is noteworthy that this variant exhibited the lowest production rate and titer in the culture supernatant. This resulted in a high degree of impurities after immobilized metal affinity chromatography, as evident by SDS-PAGE (Fig. 2A). These impurities could contribute to the observed competition, and the apparent inhibitory effect of the R101I variant may therefore be overestimated.

The impact on sC1q inhibition appears to be less for the R101S variant in the competitive interaction assay, with an IC_50_ of 0.85 µg/ml, but it is also capable of significantly inhibiting complement activation (Fig. 5). The expression and purification of the R101S variant was found to be more efficient (Fig. 2A, Table 1) compared to the R101I variant.

The R101 amino acid is located more internally, in the center of the B-chain. The exchange of arginine for isoleucine and serine results in the formation of a negatively charged spot on the protein surface (Fig. 3, B1 + 2). The R101S variant exhibits an even higher negative charge within the mutated region when compared to the R101I variant, as determined through analysis by PyMOL. Despite the fact that this amino acid has not been described as being important for IgM/C1q binding, both mutated ghC1q variants, R101I and R101S, demonstrated improved IgM/sC1q binding compared to the WT variant (Figs. 4, and 5).

In addition to the charge-based single point mutations, the N176C variant did not result in a change in the overall protein charge, but rather in the protein structure. The variant was included in the analysis because this sequence was the only one identified three times in the screened yeast display (Fig. 1). The variant demonstrated the second-highest IgM binding capacity with an IC_50_ of 0.34 µg/ml (Table 1). This effect is less pronounced in the competitive activation assay, but still significant. The mutated amino acid in the N176C variant is in close proximity to the Y175 and F178 residues at the apex of the B-chain (Fig. 3, B4), shown and proposed to be involved in IgM/C1q binding [4, 16–18]. Through the amino acid exchange, the apex of the B-chain becomes narrower, yet remains overall uncharged. It is hypothesized that this steric change enables the positively charged areas of ghC1q and the negatively charged sites of Cµ3 of IgM to come closer together, thereby strengthening the interaction between the two molecules.

Although rational protein design increased the positive surface charge of ghC1q to improve IgM binding, this could not be demonstrated. Nevertheless, the yeast display approach identified the E209R variant, which supports the hypothesis that positively charged regions are necessary for IgM binding. The importance of randomized libraries for protein engineering is confirmed by the R101 variants and the N176C variant. Further evaluation could be achieved through simulation studies or by expressing the variant as a fully mutated C1q protein, which would provide additional insights into its functional properties. Furthermore, the testing of ghC1q variants or mutated whole protein C1q proteins in cell-based assays would facilitate the acquisition of knowledge regarding this complex protein interaction and activation cascade.

### Limited expression of ghC1q variants derived from yeast surface display

In this project, we exclusively mutated the flexible loops of the ghC1q protein to avoid major changes in the framework, which could potentially compromise the expression process. As a result of the screening of the yeast surface display, we identified seven interesting variants, but only four of these variants could be successfully expressed in CHO cells. The non-expressible ghC1q variants T112K, F115K and S204E could be part of the selection background in the yeast display, indicating yeast cells that are positively transfected but do not express the ghC1q protein on the surface. Flow cytometry analysis revealed that approximately 60% of the yeast cells expressed the c-Myc tag at the C-terminus of ghC1q. A flow cytometry sorting step could be performed prior to the immunochemical enrichment steps to eliminate the 40% of non-expressing yeast cells.

Furthermore, it is possible that these proteins were expressed in the CHO system, but their presence in the culture supernatant could not be detected by quantitative biolayer interferometry or Western blot. It is evident that both methods rely on the detection of the 6xHis-tag, which may either be inaccessible or result in aggregation of the ghC1q proteins, thereby rendering the 6xHis-tag unavailable for the antibody. It has been demonstrated that there is a variability in the efficiency of detection of His-tagged proteins by anti-His antibodies [24]. In all cases, the ghC1q variants were not expressed in a form similar to the WT variant, which makes functional comparisons difficult.

### A literature review of WT ghC1q variant

The globular head of C1q expressed as a recombinant single-chain protein has been studied, both with and without different tags, and resulting from production in various expression systems. In a preceding study [8], the ghC1q variant was expressed in CHO cells with an N-terminal FLAG-tag to facilitate detection and purification. A competitive interaction assay was performed, in which coated pentameric or hexameric IgMs were used as binding partners. The FLAG-ghC1q variant achieved 65–75% inhibition in the assay using a similar hexameric IgM. This level of inhibition is higher than that achieved by ghC1q with a 6xHis-tag in the present study, which was 42% inhibition in a similar set-up. The hypothesis that the presence and position of the tag on the protein influences the inhibition capacity of the ghC1q protein is one that merits further investigation. In the competitive activation assay, the FLAG-ghC1q variant showed stronger binding to IgM than the 6xHis-tagged ghC1q, resulting in less activation by sC1q.

In the study by Vadászi et al. [25], a ghC1q variant was fused N-terminally to a 6xHis-tag and expressed in *Escherichia coli*. This ghC1q variant achieved approximately 90% inhibition at the highest concentration. However, a more detailed analysis of the materials used in the study reveals that a higher concentration of coated IgM (40 nM versus ~ 10 nM) and a tenfold higher concentration of the ghC1q variant (2000 nM versus 213 nM) were employed. At lower concentrations of ghC1q, comparable to those observed in this study, the inhibition by the 6xHis-tagged *Escherichia coli* protein was similar to the approximately 40% inhibition measured here. In summary, the format of the recombinant ghC1q protein influences its activity.

## Conclusions

In this study, we designed globular head C1q variants in a single-chain format, each differing by a single point mutation from the wild type. These ghC1q variants were developed relying on sequence alignment or model-based approaches or selected from yeast surface display. The WT and six ghC1q variants were expressed in CHO cells, purified, and tested in competitive interaction and complement activation assays.

Our results demonstrate that the ghC1q variants effectively inhibit interaction of sC1q with IgM by competing for the binding sites on IgM. The variants showed equal or greater inhibition compared to the WT, suggesting that the specific amino acid substitutions enhance inhibition potential.

This study highlights the utility of ghC1q as a tool for understanding IgM/C1q interactions and the impact of specific amino acid exchanges. It also points to the potential for developing recombinant C1q with beneficial properties for scientific and clinical applications.

## Methods

Unless otherwise stated, all chemical substances were purchased from Carl Roth.

### Protein design

The globular head C1q variants join the three globular head regions of C1q through short linkers to form a single-chain protein (human C1qA residues 110–245, UniProtKB P02745 [26]; GSG-linker; human C1qC residues 115–245, UniProtKB P02747 [27]; GSA-linker; human C1qB residues 117–253, UniProtKB P02746 [12]) [28]. The wild type variant was designed as an “originator” and used as a reference. For expression in CHO cells, a signal peptide (MDRAKLLLLLLLLLLPQAQA) [29] was N-terminally fused to all variants, and for detection and purification, the construct was linked via a GG-linker to a 6xHis-tag at the C-terminus of the B-chain. All variants of the ghC1q were generated with point mutations in the B-chain only.

### Yeast display library

The yeast display library was synthesized by GeneArt (Thermo Fisher Scientific) and cloned in the pCTcon2 plasmid (Addgene plasmid #41,843; http://n2t.net/addgene:41843; RRID: Addgene_41843 [30]). Based on the ghC1q single-chain sequence, one individual amino acid was exchanged in each variant. A total of 34 amino acid positions from the four loops of the B-chain were replaced with all remaining 19 proteinogenic amino acids, resulting in a library of 646 mutants. The plasmid library was transformed into *S. cerevisiae* EBY100 (Thermo Fisher Scientific) using PEG3350, Li-acetate and salmon sperm DNA protocol [31]. Transformed yeast cells were cultivated for 24 h at 30 °C in S-CAA basal medium supplemented with 2% glucose (SD-CAA, growth medium). For induction, the cells were diluted to OD_600_ of 1 in S-CAA medium supplemented with 2% galactose and 1% raffinose (SG/R-CAA, induction medium) and cultivated for 12 h at 20 °C. For control, transformed cells were diluted to OD_600_ of 1 in SD-CAA growth medium and cultivated for 12 h at 20 °C.

The C-terminal c-Myc fusion tag served as reporter in flow cytometry compared to not induced cells. Staining was performed in a 96-well V-bottom plate, all washing and dilution steps were done in 2% BSA-PBS buffer and centrifugation steps at 2000 g for 3 min at room temperature. In brief, cell suspension aliquots were washed and adjusted to OD_600_ of 1 before incubation with an equal volume of 1:20 diluted anti-c-Myc monoclonal antibody (clone 9E10; RRID: AB_558470; Invitrogen) at 4 °C for 30 min. After the next washing step, the cells were resuspended in an equal volume as before of 1:200 diluted goat anti-mouse Alexa Fluor 633 IgG (H + L) (RRID: AB_2535718; Thermo Fisher Scientific) and incubated at 4 °C for 30 min in the dark. After centrifugation, the cell pellet was resuspended in 100 µl buffer and measured with a CytoFLEX S (Beckman Coulter).

A plate panning approach was used to select binders. Panning was performed using a F96 Maxisorp Nunc-Immuno plate (Thermo Fisher Scientific) coated with 50 µl of 10 µg/ml hexameric IgM in carbonate coating buffer overnight at 4 °C. Before panning, the plate was washed three times with 300 µl PBS and blocked with 150 µl TBS containing 2% polyvinylpyrrolidone (PVP; Merck KGaA) for 1 h at room temperature. The induced yeast cell suspension was diluted to OD_600_ of 0.1 in TBS containing 2% PVP, 5 mM CaCl_2_ and 1.5 mM MgCl_2_. After three washes with PBS, 100 µl of the suspension was applied to the plate and incubated for 1 h at 4 °C. The wells were washed twice with PBS and filled with 150 µl of SD-CAA growth medium. The plate was sealed and incubated overnight at 30 °C. The next day, four to ten wells were pooled, transferred to a deep 24-well plate, diluted with 1.5 ml of SD-CAA media and again incubated overnight at 30 °C. For the next round of panning, cells were again induced with SG/R-CAA media for 12 h at 20 °C. After the third round of panning, 100 µl aliquots of 1:100 and 1:1000 diluted cell suspensions were plated on SD-CAA agar plates and incubated overnight at 30 °C.

For sequencing of the selected ghC1q variants after panning, 28 colonies were picked, resuspended in 50 µl of distilled water and heated at 99 °C for 5 min. 2 µl of the lysate was used as template for gene amplification.

### Recombinant cell lines

For all ghC1q variants, cDNA sequence was codon-optimized for CHO cell lines, chemically synthesized and cloned into vectors (pL vector [32] for WT, D110N and F178R variants; pcDNA3.1(+) vector for R101I, R101S, N176C and E209R variants). The corresponding recombinant CHO-K1 cell line (ATCC CCL-61) was developed as described by John et al. [8] in our laboratories.

### Protein production and purification

The recombinant proteins were produced in a semi-continuous perfusion process as described by Mayrhofer et al. [33] and John et al. [8] with BalanCD (FUJIFILM Irvine Scientific) or HyClone ActiPro (Cytiva) cell culture medium supplemented with 4 mM L-glutamine. The collected supernatants of the ghC1q variants were concentrated and buffer exchanged to equilibration buffer (20 mM sodium phosphate, 500 mM NaCl, 10 mM imidazole, pH 7.4) with a stirred cell or tangential flow filtration device (Pellicon XL cassette with Biomax membrane, NMWCO 10 kDa; Merck Millipore). Immobilized-metal affinity chromatography was performed on an ÄKTA device (Cytiva) using the HisTrap Excel column (1 ml, Cytiva). Elution was performed using a 0.3 ml/min linear gradient with elution buffer (20 mM sodium phosphate, 500 mM NaCl, 200 mM imidazole, 1 M L-arginine, pH 7.4). The eluted main fractions were pooled and buffer exchanged via Vivaspin 6 (Sartorius) or Amicon Ultra-15 (Merck KGaA) with 10 kDa MWCO to storage buffer (20 mM Tris, 150 mM NaCl) and stored at −80 °C until further use. Protein concentration was determined using biolayer interferometry-based method using HIS1K sensors in Octet RED96e (Sartorius).

### Protein modelling

Computational modelling of the ghC1q variants was performed using the PyMOL software [34] with the Adaptive Poisson-Boltzmann Solver (APBS). The globular head of the complement system protein C1q from the RCSB Protein Data Bank (PDB ID: 1PK6 [35]) was either displayed as wild type or the point mutations were introduced using the mutation tool. For the modelling using the APBS solver, always the rotamer with the highest percentage was chosen.

## Analytics

### SDS-PAGE and Western blot

The SDS-PAGE was performed using a Bolt Bis–Tris Plus Mini Protein Gel, 4–12%, 1.0 mm, and run in 1 × NuPAGE MOPS SDS Running Buffer. Reduced protein samples were prepared with NuPAGE Sample Reducing Agent. PageRuler Plus Prestained Protein Ladder was used as a molecular weight marker (all reagents from Thermo Fisher Scientific). PNGase F (New England Biolabs) was used following the denaturating protocol as recommended by the manufacturer. All protein samples were mixed with NuPAGE LDS Sample Buffer, incubated at 70 °C while shaking at 600 rpm for 10 min, and then separated on the gel at 200 V for 45 min. Staining of the gel was performed with Imperial Protein Stain (Thermo Fisher Scientific) according to the manufacturer’s instructions. Western blot was blocked and stained with a 1:1000 diluted 6xHis-tag antibody-biotin conjugate (HIS.H8-biotin, RRID: AB_2536983; Invitrogen) followed by a 1:5000 diluted streptavidin-POD conjugate (GE Healthcare) prepared with PBS buffer containing 2% BSA and 0.1% Tween-20. Membrane was developed with SuperSignal West Pico PLUS chemiluminescent substrate (Thermo Fisher Scientific) according to the manufacturer’s instructions and then analyzed with the chemiluminescent camera Fusion Fx7 device (PeqLab).

### Competitive interaction assay

The competitive interaction assay was performed as ELISA according to the protocol described by John et al. [8]. Briefly, the F96 Maxisorp Nunc-Immuno plate (Thermo Fisher Scientific) was coated with 2 µg/ml hexameric IgM diluted in carbonate coating buffer and incubated overnight at 4 °C. The plate was washed with PBS containing 0.1% Tween-20 and blocked with washing buffer containing 2% PVP. All incubation steps were performed at room temperature for 1 h with shaking. For competitive samples, low-salt TBS buffer (20 mM Tris, 50 mM NaCl, pH 7.4) with 2% PVP, 5 mM CaCl_2_ and 1.5 mM MgCl_2_ was used. The dilution series of the ghC1q variants started at 20 µg/ml and was equally mixed with a constant concentration (10 µg/ml) of human serum-derived C1q (QuidelOrtho). The mixture was applied and incubated. Detection was performed with 1 µg/ml anti-C1qC/C1qG HRP polyclonal antibody (bs-11337R-HRP; Bioss) diluted in blocking buffer. For analysis, the IC_50_ was determined using an interpolated four-parameter logistic curve, and the 50% inhibition achieved was calculated.

### Competitive complement activation assay

The competitive complement activation assay described by John et al. [8] was used with minor modifications. The F96 Maxisorp Nunc-Immuno plate (Thermo Fisher Scientific) was coated with 10 µg/ml hexameric IgM diluted in carbonate coating buffer and incubated overnight at 4 °C. The plate was washed with PBS containing 0.1% Tween-20 and blocked with washing buffer containing 2% PVP. Unless otherwise stated, all incubation steps were performed at room temperature for 1 h with shaking. Sample dilutions were made in low-salt TBS buffer (20 mM Tris, 100 mM NaCl, pH 7.4) containing 2% PVP, 5 mM CaCl_2_ and 1.5 mM MgCl_2_. Competitive samples contained 30 µl tested ghC1q variant (40 µg/ml), 30 µl 1 µg/ml serum-derived C1q (QuidelOrtho) and 60 µl 100-fold diluted C1q-depleted human serum (QuidelOrtho). The mixture was applied to the test plate and incubated for 30 min. The two-step detection was performed with goat anti-human C4 antibody (1 µg/ml, RRID: AB_2814828, Complement Technology) and rabbit anti-goat IgG peroxidase antibody (0.05 µg/ml, RRID: AB_258242, Sigma-Aldrich). Statistical significance was assessed using the Mann–Whitney U test to compare the WT variant with the respective mutant ghC1q variant.

## Data Availability

The datasets used and/or analysed during the current study are available from the corresponding author on reasonable request.

## Abbreviations

APBS: Adaptive Poisson-Boltzmann Solver
CHO: Chinese hamster ovary
C1q: Complement component 1q
ghC1q: Globular head C1q
IC_50_: Half maximal inhibitory concentration
IgG: Immunoglobulin G
IgM: Immunoglobulin M
sC1q: C1q derived from human serum
WT: Wild type
